# Feeding patterns of molestus and pipiens forms of *Culex pipiens* (Diptera: Culicidae) in a region of high hybridization

**DOI:** 10.1186/1756-3305-6-93

**Published:** 2013-04-11

**Authors:** Bruno Gomes, Carla A Sousa, José L Vicente, Leonor Pinho, Isabel Calderón, Eliane Arez, António PG Almeida, Martin J Donnelly, João Pinto

**Affiliations:** 1 Unidade de Parasitologia Médica, Instituto de Higiene e Medicina Tropical, Universidade Nova de Lisboa, Rua da Junqueira 100, Lisboa, 1349-008, Portugal; 2 Centro de Malária e outras Doenças Tropicais, Instituto de Higiene e Medicina Tropical, Universidade Nova de Lisboa, Rua da Junqueira 100, Lisboa, 1349-008, Portugal; 3 Unidade de Parasitologia e Microbiologia Médicas, Instituto de Higiene e Medicina Tropical, Universidade Nova de Lisboa, Rua da Junqueira 100, Lisboa, 1349-008, Portugal; 4 Department of Vector Biology, Liverpool School of Tropical Medicine, Pembroke Place, Liverpool, L3 5QA, UK

**Keywords:** *Culex pipiens*, Molestus, Hybridization, Host preferences, Resting behaviour

## Abstract

**Background:**

Two biological forms of the mosquito *Culex pipiens s.s.*, denoted pipiens and molestus, display behavioural differences that may affect their role as vectors of arboviruses. In this study, the feeding patterns of molestus and pipiens forms were investigated in Comporta (Portugal), where high levels of inter-form admixture have been recorded.

**Methods:**

Indoor and outdoor mosquito collections were performed in the summer of 2010. Collected *Cx. pipiens s.l.* females were molecularly identified to species and form by PCR and genotyped for six microsatellites. The source of the blood meal in post-fed females was determined by ELISA and mitochondrial DNA sequencing.

**Results:**

The distribution of the forms differed according to the collection method. The molestus form was present only in indoor collections, whereas pipiens and admixed individuals were sampled both indoors and outdoors. In both forms, over 90% of blood meals were made on avian hosts. These included blood meals taken from Passeriformes (*Passer domesticus* and *Turdus merula*) by females caught resting inside domestic shelters.

**Conclusion:**

Genetic structure and blood meal analyses suggest the presence of a bird biting molestus population in the study area. Both forms were found to rest indoors, mainly in avian shelters, but at least a proportion of females of the pipiens form may bite outdoors in sylvan habitats and then search for anthropogenic resting sites to complete their gonotrophic cycle. This behaviour may potentiate the accidental transmission of arboviruses to humans in the region.

## Background

*Culex pipiens sensu stricto* is a major vector of Japanese encephalitis serogroup arboviruses to their natural hosts, which are birds [1] and in the accidental bridge-transmission from birds to humans and domestic mammals [2,3]. This serogroup includes West Nile virus (WNV) and Usutu virus for which human cases have been reported in the European continent [4,5].

*Culex pipiens s.s.* is a synanthropic mosquito with a widespread distribution in temperate regions [6]. This species occurs as two biological forms, named molestus and pipiens, which exhibit important behavioural and physiological differences. The molestus form is stenogamous (mates in confined spaces, *i.e.* < 0.1 m^3^; [7]), autogenous (can oviposit without a blood meal), homodynamic (remains active during winter) and mammophilic (prefers to feed on mammals, including humans). In contrast, the pipiens form is eurygamous (mates in open spaces), anautogenous (oviposition requires a blood meal), heterodynamic (undergoes winter diapause) and ornithophilic (feeds predominantly on birds) [8,9].

The degree of synanthropy also varies between forms. The molestus form is more restricted to habitats with human influence, whereas the pipiens form has a greater ecological plasticity [6]. In northern temperate latitudes, molestus populations are confined to underground habitats, whereas the pipiens form occupies above ground habitats [6,10,11]. In southern Europe and in the Mediterranean region, populations of both forms occur sympatrically in aboveground habitats [12,13].

Hybridization between *Cx. pipiens s.s.* forms has been considered a major factor influencing WNV transmission [2]. Hybridization between molestus and pipiens may result in a catholic feeding behaviour thereby increasing the risk of admixed populations to act as bridge-vectors of WNV between birds and humans [14]. An influence of different molestus and pipiens genetic backgrounds on host preference has been previously documented [15]. The increase of *Cx. pipiens s.s.* bites on mammals, including humans, at the end of summer in the USA, has been attributed to a peak of hybrids in above ground habitats in this period [16]. However, a reduction of bird populations in the region at the end of the summer (specifically the American robin, *Turdus migratorius* L. 1766) may also potentiate a shift of the feeding behaviour in *Cx. pipiens s.s.*[17].

West Nile virus surveillance studies in Europe have mainly focused on the detection of the virus (or viral antigens) in natural mosquito populations [18-20]. Particular attention has also been given to the blood feeding preferences of these vector populations [21-23]. However, information about the distribution of the *Cx. pipiens s.s.* forms and hybridization rates is generally absent from these reports. More importantly, it remains to be determined how hybridization between molestus and pipiens forms can affect certain behaviours that influence pathogen transmission to humans, including blood feeding preferences, and the degree of synanthropy of the mosquito populations.

A previous study carried out in 2005-2006 in Comporta, an estuarine area in south-central Portugal, described above ground sympatric molestus and pipiens populations with incomplete genetic isolation [12]. The region is home to over 240 bird species, including migratory birds that host WNV, such as the European starling (*Sturnus vulgaris* L. 1758) and the white stork (*Ciconia ciconia* L. 1758). The interaction of the migratory birds with *Cx. pipiens s.s.* mosquitoes may establish a WNV enzootic cycle with the infection of resident WNV host birds such as the house sparrow (*Passer domesticus* L. 1758) and carrion crow (*Corvus corone* L. 1758) [24,25]. Hybridization rates of 7.6-10.3% between molestus and pipiens were recorded in this area, providing an opportunity to study the behavioural consequences of admixture between these forms [12].

In the present study, we have characterized the genetic backgrounds of molestus and pipiens in the Comporta region and related these to epidemiologically relevant traits, in particular their blood meal host preferences. Results are discussed with respect to the relative contribution of the forms, and their hybrids, to the establishment of arboviral transmission cycles.

## Methods

### Study region and mosquito collection

The Comporta region (District of Setubal, Portugal; 38° 22' 60'' N, 8° 46' 60'' W) is a wet lowland (altitude <60 m) that includes a semi-natural farming ecosystem (rice production and cork-oak forest) and a protected landscape, the national wildlife reserve of Estuário do Sado. The region has a warm temperate climate with hot dry summers and mild winters (class Csa, Köppen Classification System [26]) with monthly averages of mean daily temperature varying between 10°C and 21°C and daily rainfall between 0.12 and 3.4 mm.

Mosquito collections took place over two weeks in 2010 (19^th^ - 23^rd^ July and 7^th^ -13^th^ August) in 7 localities of the region (Additional file 1: Table S1). Three sampling methods were used: i) indoor resting collections (IR) were performed inside domestic animal shelters using hand mechanical aspirators and torches. Each animal shelter was inspected for mosquitoes for a period of 10 min; ii) outdoor CDC light trap (Centers for Disease Control [27]) collections, placed in the canopy of trees (CDC-C) and at ground level (CDC-G), were performed overnight between 19:00-09:00; iii) outdoor human landing catches (HLC) were performed between 20:00-23:00 by a team of four collectors using hand mechanical aspirators and torches.

Collected mosquitoes were killed by freezing and identified to species/complex using morphological keys [28]. Freshly blood-fed female mosquitoes obtained by indoor resting and CDC light trap collections had their abdomens removed and preserved in 20 μl EDTA (0.125 M) at -20°C for subsequent blood meal identification. The thorax and head of each blood-fed female was preserved individually at -20°C until DNA extraction. Non blood-fed whole mosquitoes were preserved in the same conditions as the heads and thoraces.

### Mosquito DNA extraction and molecular analysis

DNA was extracted from individual females (whole body or head plus thorax) using a phenol-chloroform method with ethanol precipitation [29]. Each specimen was identified to species by a multiplex PCR assay targeting species-specific polymorphisms in the intron-2 of the acetylcholinesterase-2 (*ace*-*2*) gene using primers specific for *Cx. pipiens s.s., Cx. quinquefasciatus* and *Culex torrentium*[30].

### Selection and analysis of microsatellite loci

The software WHICHLOCI [31] was applied to the microsatellite dataset used by Gomes *et al.*[12] to determine the genetic backgrounds of molestus and pipiens in Comporta, in order to select a subset of six loci to be analysed in this study. Of the 13 microsatellites that were genotyped in Gomes *et al.*[12], locus CQ11 was excluded due to its linkage with the diagnostic CQ11FL marker (see below). The remaining 12 microsatellite datasets were used to create three samples of 500 simulated individuals (molestus, pipiens and hybrids) to infer, under 10^5^ iterations, which combinations of microsatellites allow correct assignment of the simulated individuals with a minimum accuracy of 90%. Bayesian clustering analysis, as implemented by STRUCTURE 2.3.3. [32], was then used to infer population structure in the data set of Gomes *et al.*[12] with the best six microsatellites and under the same run conditions. The results obtained for the datasets with six and 13 microsatellites were compared to establish the robustness of the analysis with the lowest battery of microsatellite loci (*i.e.* six).

Microsatellite genotyping was performed by PCR with fluorescently-labelled primers under the same conditions as in Gomes *et al.*[12]. Amplified products were separated by capillary electrophoresis in a genetic analyser ABI3730 (Applied Biosystems), at Yale DNA Analysis Facility (USA). Fragment sizes and genotypes were scored using the software GeneMarker 1.4. (Softgenetics, USA).

The multiplex PCR assay described by Bahnck & Fonseca [33] was used to detect a size polymorphism in the 5' flanking region of the CQ11 microsatellite of *Cx. pipiens s.s.* that differentiates molestus and pipiens forms as well as their hybrids. This marker, here denoted as CQ11FL, differentiates specimens of the pipiens form (200 bp or 350 bp) from the molestus form (250 bp PCR product) while hybrids exhibit both pipiens and molestus amplicons [33,34]. Given its relatively good performance at the population level in the region [12], this marker was used to label distinct microsatellite-based genetic clusters as belonging to the molestus or pipiens forms.

### Blood meal identification

A Sandwich ELISA protocol [35] was used to identify blood meals of blood-fed indoor resting mosquitoes. Blood meals were tested for the presence of chicken, cow, dog, goat/sheep, horse/donkey, human, pig, and rabbit immunoglobulin G (IgG). Four positive controls (blood from the tested species) and 14 negative controls (two blood samples from the other seven species) were used in every 96-well microplate. Absorbance values were read at 492 nm wave length in an ELISA reader (Anthos 2010 ®, Anthos Labtec Instruments). Cut-off values were calculated for each plate, as the mean plus three times the standard deviation of the negative controls.

Fragments of the mitochondrial DNA *cytochrome b* (*cyt b*) gene were sequenced to identify the blood meal source of female mosquitoes collected in the canopy of trees (CDC-C) and for a subsample of females caught indoor resting (ELISA-negative blood meals and random blood meals from all the different types of blood meal identified). DNA extraction from blood samples was performed with the DNeasy Blood &Tissue Kit (Qiagen, Valencia, CA). The vertebrate *cyt b* gene was amplified following a modified version of the protocol of Hamer *et al.*[36] that excluded the fourth primer pair amplification. PCR products were purified with the QIAquick PCR Purification kit (Qiagen) and sequenced in a biotechnology company (StabVida, Oeiras) on an ABI3730XL automated sequencer (Applied Biosystems). Sequences were manually corrected and aligned using BioEdit 7.0.9.0 [37]. Identification of host species was performed by comparison with *cyt b* sequences deposited at NCBI GenBank.

### Data analysis

Bayesian clustering analysis, as implemented by STRUCTURE 2.3.3. [32], was used to infer population substructure/ancestry from the data set without prior information of sampling groups under the conditions of admixture (*α* allowed to vary between 0 and 10), and allele frequencies correlated among populations (*λ* was set at 1, default value). Ten independent runs with 10^4^ iterations and 10^5^ replications were performed for each value of *K* (*K* = 1 to 10 clusters). To infer the most likely number of clusters in the sample, the *ΔK* statistic was used [38]. Information from the outputs of each *K* (10 runs) was compiled by the Greedy method implemented in CLUMPP [39]. Following the suggestions of Vähä & Primmer [40], individual genetic assignment to clusters was based on a minimum posterior probability threshold (*Tq*) of 0.90. Individuals displaying 0.1≤ *q*_*i*_ ≤0.90 were considered of admixed ancestry.

Genetic diversity at each microsatellite locus was characterised by estimates of expected heterozygosity (*H*_*e*_) [41] and inbreeding coefficient (*F*_*IS*_). Significance of *F*_*IS*_ values was assessed by randomisation tests. These analyses were performed using FSTAT v. 2.9.3.2. [42]. Estimates of allele richness (*A*_*R*_), adjusted for the lowest sample size, were obtained by a rarefaction statistical approach implemented by the programme HP-RARE [43]. Departures from Hardy–Weinberg equilibrium were tested by exact tests available in ARLEQUIN v.3.5. [44]. The same software was used to perform exact tests of linkage equilibrium between pairs of loci based on the expectation-maximisation approach described by Slatkin & Excoffier [45]. The software MICRO-CHECKER 2.2.3. was used to search (99% confidence interval) for null alleles at loci/samples [46].

Fisher’s exact tests (2×2) were performed with *“VassarStats: Website for Statistical Computation”*[47] to determine associations between the genetic clusters identified by STRUCTURE and the origin of blood meals.

Whenever multiple testing was performed, the nominal significance level of rejection of the null hypothesis (*α* = 0.05) was corrected by the sequential Bonferroni procedure [48].

## Results

### Mosquito sampling

A total of 80 IR collections were performed in 28 sites (Table 1). The majority of animal shelters found in the area were chicken coops (46.4%). Consequently, 44 (55.0%) of the IR collections were made in chicken coops, whereas 19 (23.8%) were made in shelters harbouring mammalian hosts without domestic birds (*i.e.* rabbit hutches, cattle barns and pig pens). Seven (8.8%) collections were performed in shelters with both avian and mammalian hosts and 10 (12.5%) inside installations without any visible vertebrate host. The IR collections yielded a total of 235 *Cx. pipiens s.l.* females, of which 174 (74.0%) were blood fed. Of the total of females caught, 88.5% were sampled inside chicken coops, 4.3% in mammalian shelters, 3.8% in mixed avian-mammal shelters and 3.4% in installations with no domestic vertebrates (Table 1). None of the 10 females caught inside shelters exclusively with mammalian hosts was blood fed and only 6 (3.4%) engorged females were collected in mixed avian-mammalian shelters.

**Table 1:** **Number of indoor resting collections and *Cx. pipiens s.l. *mosquitoes caught according to the type of shelter**

**Shelters**	**IR sites**	**Collections**		***Cx. pipiens s.l.***	
		***N***_***C***_	***N***_***PC***_	***N***_***F***_	***N***_***BF***_
Chicken coops	13	44	31	208	164
	(46.4)	(55.0)	(70.5)	(88.5)	(94.3)
Rabbit hutches	3	11	4	10	0
	(10.7)	(13.8)	(9.1)	(4.3)	(0.0)
Cattle barns	1	4	0	0	0
	(3.6)	(5.0)	(0.0)	(0.0)	(0.0)
Pig pens	3	4	0	0	0
	(10.7)	(5.0)	(0.0)	(0.0)	(0.0)
Mixed composition	3	7	5	9	6
	(10.7)	(8.8)	(11.4)	(3.8)	(3.4)
Without vertebrates	5	10	4	8	4
	(17.9)	(12.5)	(9.1)	(3.4)	(2.3)
Total	28	80	44	235	174

IR sites: number of indoor resting collection sites surveyed; *N*_*C*_: number of collections performed; *N*_*PC*_: number of collections positive for *Cx. pipiens s.l.*; *N*_*F*_: number of *Cx. pipiens s.l. *females collected; *N*_*BF*_: number of blood-fed females collected; Mixed composition: Shelter with domestic birds and domestic mammals. Values in brackets represent relative frequencies (in percentage).

A total of 24 outdoor CDC light trap collections were performed (Additional file 1: Table S1). Of these, 17 were performed with traps hung in the canopy of trees (CDC-C), yielding 1,093 *Cx. pipiens s.l.* females, and 7 were placed at ground level yielding a total of 625 females. Human landing catches were performed six times at a single site (Additional file 1: Table S1). These collections yielded a total of 155 *Cx. pipiens s.l.* females. The mean number of bites per human per hour was 2.2 for this species.

### Microsatellite analysis

The best combination of six microsatellites (assigned score of 92.0%) included loci CxpGT04, CQ26, CxpGT20, CxpGT12, CQ41, and CxpGT40 (Additional file 1: Table S2). The analysis with six loci was able to split the Gomes *et al.*[12] dataset into two clusters with a highly similar result to that obtained with 13 loci (Additional file 1: Table S3). Taking the 13 loci dataset as the gold standard, the analysis with six loci had an average accuracy (*i.e.* average of the number of correctly identified individuals for a class over the total number of individuals assigned to that class) of 81.6% and average power (*i.e.* average of the number of correctly identified individuals for a class over the actual number of individuals of that class) of 88.6%.

Of the IR collections, only blood-fed females caught inside shelters with vertebrate hosts were selected for molecular genotyping (*N* = 174). Of these, four specimens failed in PCR amplifications and were thus excluded. A total of 170 females from five of the seven localities (Cambado: *N* = 14; Comporta: *N* = 27; Pego: *N* = 47; Possanco: *N* = 80; Torre: *N* = 2) were analysed. In addition to IR mosquitoes, subsamples from CDC-C (*N* = 39, of which 9 were blood fed), CDC-G (*N* =42) and HLC (*N* = 40) were also included, giving a total of 291 specimens used for molecular identification and microsatellite genotyping. All specimens were molecularly identified as *Cx. pipiens s.s.* by PCR [30].

**Figure 1:**
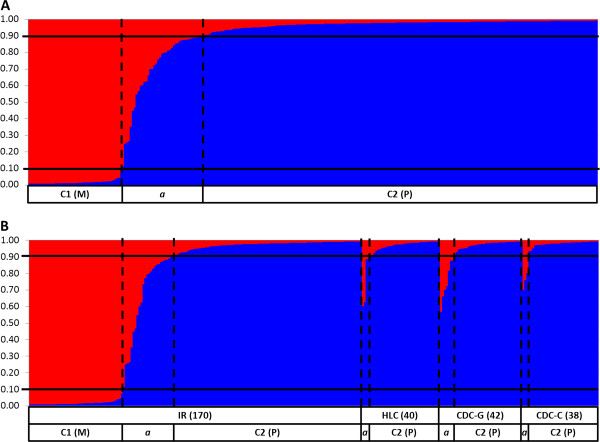
**Bayesian cluster analysis of *Cx. pipiens s.s. *mosquitoes conducted by STRUCTURE in Comporta (2010). A**: Individuals sorted by their ancestral probability; **B**: Individuals sorted by collection method and ancestral probability; IR: indoor resting inside shelters; CDC-G: CDC light traps in ground level; CDC-C: CDC light traps in canopy of trees; HLC: human landing catches; *a:* admixed individuals (0.1 < *Tq* < 0.9). Columns correspond to the multilocus genotype of each individual, partitioned in different colours representing the probability of ancestry (*q*_*i*_) to each cluster (Red: molestus; Blue: pipiens). Individuals were ordered according to their geographic information. Lines indicate the *q*_*i *_threshold used to determine admixed individuals (see Methods).

Bayesian clustering analysis implemented by STRUCTURE revealed two clusters (Figure 1A). Cluster 1 grouped 48 specimens of which 42 (87.5%) were classified as molestus form by the CQ11FL locus (Table 2). The majority (84.3%) of the 204 specimens in cluster 2 was classified as pipiens form by CQ11FL (Table 2). There were 39 females exhibiting an admixed ancestry (*i.e. q*_*i*_ ≥ 0.10 for both clusters). Of these, seven (17.9%) had a heterozygous CQ11FL200/250 genotype while the majority (*N* = 31, 79.5%) were classified as pipiens form by CQ11FL PCR (Table 2). There were twelve individuals displaying a 350 bp CQ11FL allele. Of these, 11 were grouped in the pipiens cluster, while one CQ11FL200/350 heterozygote was assigned to the admixed cluster (Table 2).

**Table 2:** Frequencies of molecular identification at the CQ11FL in each of the ancestry clusters revealed by STRUCTURE

	***N***	**CQ11FL genotype**		
		**250/250**	**200/250**	**200/200**
Cluster 1 (molestus)	48	42	2	4
		(87.5)	(4.2)	(8.3)
Cluster 2 (pipiens)	204^a^	6	25^b^	172^c^
		(2.9)	(12.3)	(84.3)
Admixed	39	1	7	31^d^
		(2.6)	(17.9)	(79.5)
Total	291	49	34	207
		(11.7)	(16.8)	(71.1)

*N*: number of individuals; Values in parenthesis refer to the frequencies (in percentage) within each cluster. ^a ^includes one specimen without CQ11FL identification. ^b ^includes one CQ11FL250/350 heterozygote. ^c^ includes one CQ11FL350/350 homozygote and nine CQ11FL200/350 heterozygotes. ^d^ includes one CQ11FL200/350.

Genetic diversity estimates for the 6 microsatellite loci analysed for the whole dataset (*N* = 291) and in subsamples determined by clustering analysis (STRUCTURE) and by sampling type (*i.e.* collections inside animal shelters versus outdoor collections) are shown in Table S4 (see Additional file 1). Significant departures from Hardy-Weinberg equilibrium were detected at 5 loci (83.3%) when all specimens were analysed as a single sample (Additional file 1: Table S4). However, when the sample was subdivided according to clustering assignment and sampling site, significant heterozygote deficits were observed only on six occasions (21.4% out of 28 tests). These departures were generally associated with significant positive *F*_*IS*_ values indicative of a heterozygote deficit. Exact tests of linkage disequilibrium revealed 12 (80.0%) significant associations between pairs of loci for the whole dataset. When samples were divided by clustering assignment and type of sampling site, only one significant association was observed (1.3% out of 75 combinations). The analysis performed by MICRO-CHECKER did not find a consistent signal of null alleles in any loci. All microsatellite loci were maintained for subsequent analyses.

Bayesian clustering analysis showed a non-uniform distribution of the forms among collection methods (Figure 1B). All specimens with a molestus genetic background were sampled solely by IR collections, whereas individuals with pipiens or admixed ancestry were collected by both IR and outdoor collections (*i.e.* CDC-C, CDC-G and HLC). In IR collections, the proportion of molestus individuals caught inside chicken coops was 28.8% of the total catch and 16.7% in avian-mammal mixed shelters. The proportion of admixed individuals caught by IR (14.7%, 25 out of 170) was comparable to that sampled by outdoor collection methods (11.6%, 14 out of 121).

**Figure 2:**
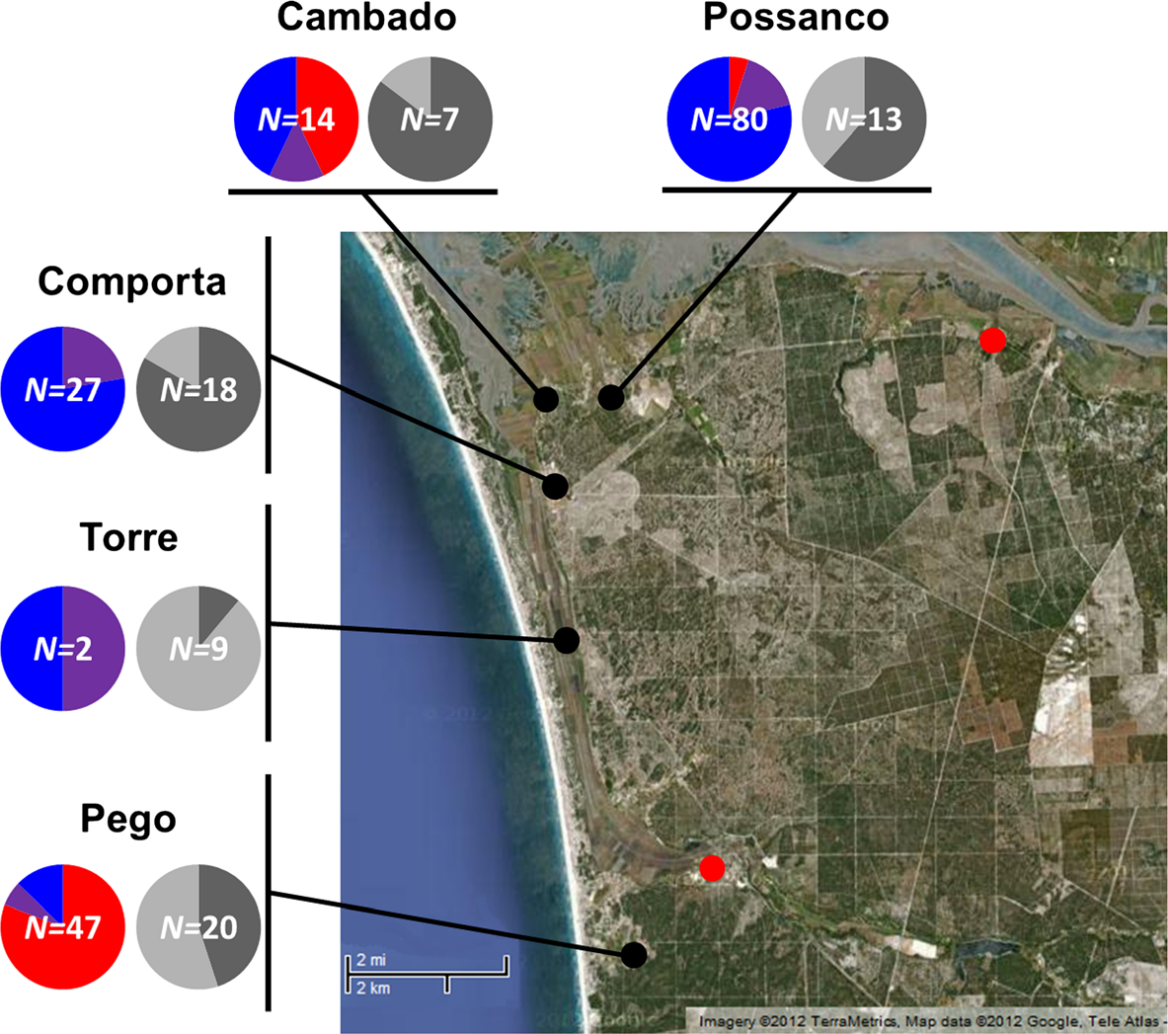
**Frequency of the groups defined by STRUCTURE by locality. **Black dot: positive sample site for *Cx. pipiens s.s. *(Cambado, Comporta, Pego, Possanco, Torre); Red dot: negative sample site for *Cx. pipiens s.s. *(Carvalhal, Monte Novo do Sul). Color graphics: proportion of females; Red: cluster 1 (molestus form); Blue: cluster 2 (pipiens form); Purple: *admixed *(hybrids). Grey-scale graphics: proportion of mosquito collections; Dark grey: proportion of collection performed in chicken coops; Light grey: proportion of collection performed in other type of shelter.

The distribution of *Cx. pipiens s.s.* forms in IR collections appeared not to be homogenous among the localities surveyed (Figure 2). Individuals of molestus ancestry were concentrated mainly in Pego (79.2%), constituting 80.9% of the total IR catch at this locality. The proportion of IR collections made at avian shelters (*i.e.* chicken coops) in Pego was 45.0%, whereas it varied between 61.5% and 83.3% in the two localities where the pipiens form predominated (Comporta and Possanco; Figure 2).

### Blood meal identification

Blood meal identification by ELISA revealed that most (*N* = 159; 93.5%) of the 170 blood feeds analysed were from avian hosts (Table 3). The proportion of blood meals taken on avian hosts by pipiens (95.9%) and molestus (91.6%) forms was not significantly different (Fisher’s exact test: *P* = 0.108; Table 3). All admixed individuals fed on avian hosts. There were only three single blood meals taken on mammalian hosts. All consisted of human blood taken by two molestus and one pipiens females. There were also two females (one molestus and one admixed) with a mixed blood meal with cow and avian blood. The ELISA did not identify the origin of six blood meals (three pipiens, two molestus and one hybrid).

**Table 3:** Blood meal source identification (by ELISA) in each of the ancestry clusters revealed by STRUCTURE in indoor collections

	***N***	**Blood feed – Indoor**			
		**Mammal**	**Bird**	**Mixed**	**WI**
Cluster 1 (molestus)	48	2	43	1	2
		(4.2)	(89.5)	(2.1)	(4.2)
Cluster 2 (pipiens)	97	1	93	0	3
		(1.0)	(95.9)	(0.0)	(3.1)
Admixed	25	0	23	1	1
		(0.0)	(92.0)	(4.0)	(4.0)
Total	170	3	159	2	6
		(1.8)	(93.5)	(1.2)	(3.5)

*N*: number of individuals; Mammal: feeds in mammal (all in Human); Bird: feeds in Bird (chicken antibody); Mixed: Mixed fed in mammal and bird (all in cow and chicken); WI: without positive identification. Values in parenthesis refer to the frequencies (in percentage) within each cluster.

Sequence analysis of the *cyt b* gene in blood samples was performed for the nine engorged females caught in light traps from the tree canopy (CDC-C) and 19 specimens from IR collections (the six females without ELISA identification, two females with mixed feeds, three females with only mammalian blood, and eight females with only avian blood). Two samples did not amplify *cyt b* gene of any vertebrate (one molestus female without ELISA identification and one female from the canopy). The *cyt b* analysis confirmed the ELISA results for the females with single feed but identified only chicken (*Gallus gallus* L. 1758; GenBank: DQ512918.1) mtDNA in the blood of the two females with mixed feeds. Two bird species were identified in the five females caught IR without ELISA identification: house sparrow (*Passer domesticus*; GenBank: AY495393.1) in four females (two pipiens, one molestus and one hybrid), and blackbird (*Turdus merula* L. 1758; GenBank: EU154637.1) in one pipiens female. In the nine females collected by CDC-C, two other bird species were identified: long-eared owl (*Asio otus* L. 1758; GenBank: AF082067.2) blood in eight females (seven pipiens and one hybrid) and blue tit (*Cyanistes caeruleus* (L. 1758); GenBank: AF347961.1) blood in one pipiens female.

## Discussion

In this study, a notable difference was found in the distribution of molestus and pipiens forms according to collection methods. While the pipiens form was sampled by all methods, molestus individuals were caught only in IR collections. This result suggests differences between forms in biting and resting behaviours. When placed outdoors, CDC light traps are appropriate for sampling both host seeking mosquitoes and recently blood-fed mosquitoes searching for a suitable resting site [49]. These traps have been successfully used as an alternative to outdoor resting collections in feeding pattern studies of *Cx. pipiens s.l.* conducted in the USA [17,50]. The absence of the molestus form from outdoor CDC light trap collections may suggest a more endophagic and endophilic behaviour of this form. A tendency of the molestus form to bite indoors was further highlighted by its absence from outdoor landing catches. These results point to a predominantly indoor and synanthropic behaviour of the molestus form, as described for populations of this form at northern latitudes where inter-form hybridization is rare [6,10,16]. Therefore, it appears that in spite of the high hybridization levels and in addition to autogeny and stenogamy, the molestus population of Comporta maintains behavioural phenotypes typical of this form. This observation is consistent with a pure molestus genetic background found in the region, which contrasted with a more introgressed pipiens background [12].

Also compatible with a pattern of asymmetric hy-bridization, with more molestus genes introgressing the pipiens form, is an apparently more plastic resting behaviour of the pipiens form, suggested by the fact that blood-fed females of this form were collected both indoors and outdoors. However, the number of blood-fed *Cx. pipiens s.s.* females collected in outdoor CDC light traps (9 out of 1,718) was much lower than those in IR collections (174 out of 235). Furthermore, the apparent behavioural differences observed between pipiens and molestus forms should be considered with caution given the sampling design used in this study, which did not include paired collections with the same method. Additional surveys involving paired indoor/outdoor landing catches (to directly evaluate endo/exophagy) and indoor/outdoor resting collections would be required to confirm these observations.

The approach used for the selection of microsatellites to differentiate molestus and pipiens forms allowed reduction of the number of loci to be genotyped from 13 to 6 whilst maintaining high accuracy and power. The efficiency of multilocus analyses tends to increase with the number of microsatellite [40]. However, the use of a more limited number of loci can benefit their application in surveillance studies by minimising genotyping costs and thus allowing genotyping of larger sampling sizes. Given the importance of accurately determining the intra-specific composition of *Cx. pipiens s.s.* it is recommended that similar microsatellite-based approaches are used in epidemiological surveys to complement the information based on a single marker (CQ11FL) that has limitations in areas of continued introgression [12,33].

As in the survey conducted in 2005-2006 [12], sympatric molestus and pipiens populations displaying high hybridization levels were identified above ground in the region of Comporta. However, a higher proportion of the molestus form was found in a 2005-2006 survey (66.2%; Gomes *et al.,* 2009), whereas in the present study the pipiens form prevailed (70.1%). This difference most likely reflects the outdoor sampling carried out in this study and which was not carried out in the previous survey. In addition, the survey of 2005/2006 was mainly concentrated in the locality of Pego (*ca.* 77% of females), where 79% of molestus individuals were collected in the present survey. The reasons for a higher frequency of the molestus form in Pego remain unclear. Although the proportion of chicken coops sampled in this locality was lower than in those localities where the pipiens form predominated, this may not explain the differences since the vast majority (>90%) of *Cx. pipiens s.s.* was sampled inside chicken coops in the three localities where the molestus form was detected. Other factors may affect the apparently heterogeneous distribution of the forms, such as the type of animal shelters (*e.g.* construction materials) or differences in breeding site availability and exploitation. More detailed ecological studies are thus needed to further clarify the presence and determinants of spatial heterogeneities between forms in this region.

Blood meal analysis revealed that the great majority of *Cx. pipiens s.s.* females fed on avian hosts. The pipiens form showed a slightly higher proportion of avian blood feeds when compared with the molestus form. However, this difference was non-significant and the proportion of avian blood meals was above 90% in both forms, suggesting an ornithophilic tendency for *Cx. pipiens s.s.* in the region. Ornithophilic tendency was also observed in a study analysing *Cx. pipiens s.l.* from urban and countryside areas of Portugal, in which over 70% of the females fed on birds [23], and from south-west countryside areas of Spain, where over 80% of the females fed on birds [22]. The pipiens form has been described as ornithophilic, whereas molestus populations were recognised as being mammophilic [8,9]. However, the feeding patterns of *Cx. pipiens s.s.* populations depend not only on their genetic background but also on the availability of vertebrate hosts and on host defensive mechanisms [15,21]. Consequently, exceptions to the general feeding pattern have been reported for both forms in the USA and in the Mediterranean region [15,51,52]. Furthermore, hybridization between the two forms may also promote a more opportunistic feeding behaviour in *Cx. pipiens s.s.*[2]. Such a catholic behaviour would thus increase the relative importance of host availability and host defensive mechanisms in the feeding pattern of the mosquito population. Therefore, continued hybridization between forms coupled with a greater availability of avian hosts in the study area may explain the greater proportion of avian feeds taken by the molestus population, which is otherwise considered as being mammophilic.

While molestus and pipiens appear to be mainly ornithophilic in the Comporta region, this may reflect host availability in the region rather than an intrinsic host preference. A lower availability of mammals (including humans) is suggested by a higher proportion of chicken coops (46.4%) when compared to mammalian shelters without domestic birds (25.0%), and by the well-built and protected human dwellings with door and window screens that prevent mosquito entry [53]. On the other hand, pipiens form mosquitoes were caught biting humans outdoors in HLC collections which play in favour of a more opportunistic feeding pattern promoted by hybridization. Altogether, these findings suggest a closer association of both molestus and pipiens forms with avian hosts and that this ornithophilic tendency, albeit possibly genetically conditioned, may be mainly determined by host availability in the region. In this scenario, molestus females, with a preference for biting mammalian hosts, may feed more readily on the available bird hosts, which may increase the odds for alternate feeding on birds and mammals. This feeding behaviour may increase the risk of WNV transmission from birds, which are natural amplification hosts, to accidental hosts such as humans and domestic mammals.

It is worth noting, however, that the two mosquito collections carried out in this study took place in mid-summer. This did not allow inference on the possible seasonal variations in the feeding patterns of the forms, which may vary through time due to factors such as temperature and bird migration [17]. Further studies, involving longitudinal sampling, will be required to further clarify the intrinsic host preference of *Cx. pipiens* forms in the region and if host availability is the main factor modulating the feeding patterns over time, in a similar manner to what has been observed in the USA [17].

Blood meal host identification based on mtDNA sequencing identified bird species from Passeriformes and Strigiformes orders. Birds from these orders were identified with anti-WNV antibodies in Portugal indicating the circulation of WNV in these populations [54]. The Passeriformes are a well-known WNV reservoir [55,56]. Species of this order, such as *Passer domesticus*, displayed the highest WNV prevalence in USA [36,57].

## Conclusion

The presence of females from both forms collected inside domestic animal shelters with a blood meal taken from wild Passeriformes gives a clear indication of the proximity between the WNV natural cycle and the human population in the Comporta region. Species such as the house sparrow and the blackbird have tolerance for humans and the blood meal could have been taken indoors when those birds enter in human constructions searching for food or shelter. However, the combination of the genetic structure and blood meal analysis suggest that at least a proportion of pipiens form females may bite outdoors in sylvan habitats and then search for anthropogenic indoor resting sites to complete their gonotrophic cycle. In both scenarios, alternative domestic hosts and humans are available in those sites for subsequent blood feeding, which may promote the accidental transmission of WNV and other arboviruses in this region.

## Abbreviations

ace-2: acetylcholinesterase-2 gene; AR: allelic richness; bp: base pair; ca.: *circa*; CDC-C: Centers for Disease Control light trap placed in the canopy of trees; CDC-G: Centers for Disease Control light trap placed at ground level; CQ11FL: molecular assay in the 5' flanking region of the CQ11 microsatellite; Csa: temperate climate with dry and hot summers; Cx.: *Culex*; cyt b: *cytochrome b*; DNA: deoxyribonucleic acid; dNTPs: deoxynucleotide triphosphates; EDTA: ethylenediaminetetraacetic acid; ELISA: enzyme-linked immunosorbent assay; et al.: *et alli*; FIS: inbreeding coefficient; FST: fixation index; i.e.: *id est*; IgG: immunoglobulin G; He: expected heterozygosity; HLC: human landing catches; IR: indoor resting collections; K: number of clusters; L.: Linnaeus; min: minutes; mtDNA: mitochondrial deoxyribonucleic acid; N: sample size; NCBI: National Center for Biotechnology Information; P: probability; PCR: polymerase chain reaction; qi: probability of ancestry; s.l.: *sensu lato*; s.s.: *sensu stricto*; Tq: posterior probability threshold; USA: United States of America; WNV: West Nile virus; α: nominal significance level of rejection of the null hypothesis; ΔK: *ad hoc* approach to infer the most likely number of clusters in the sample by the STRUCTURE.

## Competing interests

The authors declare that they have no competing interests.

## Authors’ contributions

BG, CAS, JLV, LP and IC carried out sample collections. Molecular analyses were conducted by BG, JLV and EA. BG and JP performed the genetic data analysis. BG, CAS, APGA, MJD and JP conceived the study and designed the experiments. BG and JP drafted the manuscript with the contributions of CAS, APGA and MJD. All authors read and approved the final manuscript.

## Supplementary Material

Additional file 1: Table S1 – Localities surveyed, number of sites sampled and number of collections performed for each collection method. **Table S2 **– Loci ranking performed by WHICHLOCI with 12 microsatellites. **Table S3 **- Accuracy and power of the clustering analysis performed by STRUCTURE [32] with 6 loci for the 13 microsatellites dataset of Gomes et al. [12]. **Table S4 **– Genetic diversity at microsatellite loci of *Culex pipiens s.s. *from Comporta. Click here for file
