# Supplementary material for: Feeding patterns of molestus and pipiens forms of Culex pipiens (Diptera: Culicidae) in a region of high hybridization
Source: Parasit Vectors. 2013 Apr 11;6:93. doi: 10.1186/1756-3305-6-93 (PMC3637809; doi:10.1186/1756-3305-6-93)
Supplement: Additional file 1: Table S1 — – Localities surveyed, number of sites sampled and number of collections performed for each collection method. Table S2 – Loci ranking performed by WHICHLOCI with 12 microsatellites. Table S3 - Accuracy and power of the clustering analysis performed by STRUCTURE [32] with 6 loci for the 13 microsatellites dataset of Gomes et al. [12]. Table S4 – Genetic diversity at microsatellite loci of Culex pipiens s.s. from Comporta. [file 1756-3305-6-93-S1.docx]

Table S1 – Localities surveyed, number of sites sampled and number of collections performed for each collection method

| **Locality** | **latitude** | **longitude** | **Collection method** | | | | | | | |
| --- | --- | --- | --- | --- | --- | --- | --- | --- | --- | --- |
|  |  |  | **IR** | | **CDC-C** | | **CDC-G** | | **HLC** | |
| Cambado | 38°23'39"N | 8°47'16"W | 3 | (7) | 2 | (4) | 1 | (1) | 0 | (0) |
| Carvalhal | 38º18'35"N | 8º45'05"W | 2 | (6) | 3 | (3) | 1 | (1) | 0 | (0) |
| Comporta | 38º22'42"N | 8º46'59"W | 7 | (18) | 2 | (3) | 0 | (0) | 1 | (6) |
| Monte Novo do Sul | 38°24'28"N | 8°40'53"W | 4 | (7) | 1 | (1) | 1 | (1) | 0 | (0) |
| Pego | 38°17'36"N | 8°46'05"W | 4 | (20) | 1 | (2) | 1 | (1) | 0 | (0) |
| Possanco | 38°23'48"N | 8°46'23"W | 4 | (13) | 2 | (2) | 1 | (1) | 0 | (0) |
| Torre | 38°21'09"N | 8°46'50"W | 4 | (9) | 1 | (2) | 2 | (2) | 0 | (0) |
| **Total** |  |  | 28 | (80) | 12 | (17) | 7 | (7) | 1 | (6) |

IR: number of sites (shelters) sampled by indoor resting collections; CDC-C: number of sites sampled by CDC light traps in canopy of trees; CDC-G: number of sites sampled by CDC light traps at ground level; HLC: number of sites sampled by human landing catches. Values in parenthesis refer to the number of collections performed.

Table S2 – Loci ranking performed by WHICHLOCI with 12 microsatellites

| **Rank** | **Locus** | **Score** | **Score (%)** | **A (%)** |
| --- | --- | --- | --- | --- |
| 1 | **CxpGT04** | 0.688 | 10.27 | 92.00 |
| 2 | **CQ26** | 0.661 | 9.87 |  |
| 3 | **CxpGT20** | 0.660 | 9.85 |  |
| 4 | **CxpGT12** | 0.655 | 9.78 |  |
| 5 | **CQ41** | 0.649 | 9.69 |  |
| 6 | **CxpGT40** | 0.618 | 9.22 |  |
| 7 | CxpGT51 | 0.557 | 8.31 | NA |
| 8 | CxqTri4 | 0.513 | 7.65 |  |
| 9 | CxqGT6b | 0.483 | 7.20 |  |
| 10 | CxpGT53 | 0.463 | 6.90 |  |
| 11 | CxpGT46 | 0.403 | 6.01 |  |
| 12 | CxqGT4 | 0.351 | 5.24 |  |

A: correct assignment with 6 loci; NA: Not applicable.

Table S3 - Accuracy and power of the clustering analysis performed by STRUCTURE [32] with 6 loci for the 13 microsatellites dataset of Gomes *et al.* [12]

|  | **Golden standard**  **(13 loci)** | **Assigned**  **(6 loci)** | **Correctly assigned**  **(6 loci)** | **Power** | **Accuracy** |
| --- | --- | --- | --- | --- | --- |
| **Cluster 1 (molestus)** | 96 | 88 | 88 | 0.916 | 1.000 |
| **Cluster 2 (pipiens)** | 36 | 38 | 35 | 0.972 | 0.921 |
| **hybrids** | 13 | 19 | 10 | 0.769 | 0.526 |

Power: number of correctly identified individuals for a class over the actual number of individuals of that class; Accuracy: number of correctly identified individuals for a class over the total number of individuals assigned to that class. Individual assignment was based on a *Tq*>0.9, hybrids: 0.1<*Tq*<0.9.

Table S4 – Genetic diversity at microsatellite loci of *Culex pipiens* s.s. from Comporta

| Locus |  | Inside animal shelters | | |  | Outdoor | |  | Total  (*N*=291) |
| --- | --- | --- | --- | --- | --- | --- | --- | --- | --- |
|  |  | P  (*N*=98) | H  (*N*=25) | M  (*N*=48) |  | P  (*N=*107) | H  (*N=*14) |  |  |
| CQ26 | *A_R(26)_* | 6.4 | 7.1 | 6.0 |  | 7.4 | 6.9 |  | 7.7 |
|  | *H_e_* | 0.768* | **0.820*** | **0.765*** |  | 0.813 | 0.804 |  | **0.825** |
|  | *F_IS_* | 0.151 | **0.370** | **0.349** |  | 0.078 | 0.207 |  | **0.209** |
| CQ41 | *A_R(26)_* | 9.8 | 9.7 | 6.4 |  | 10.3 | 8.8 |  | 10.5 |
|  | *H_e_* | 0.839* | 0.840 | 0.795 |  | **0.842*** | 0.817 |  | **0.853** |
|  | *F_IS_* | **0.163** | 0.145 | -0.098 |  | **0.273** | 0.220 |  | **0.184** |
| CxpGT04 | *A_R(26)_* | 8.7 | 7.3 | 3.3 |  | 8.7 | 8.9 |  | 8.5 |
|  | *H_e_* | 0.864 | 0.735 | 0.573 |  | 0.865 | 0.857 |  | 0.836 |
|  | *F_IS_* | -0.014 | -0.091 | -0.277 |  | -0.005 | 0.000 |  | -0.008 |
| CxpGT12 | *A_R(26)_* | 7.0 | 4.7 | 2.3 |  | 6.9 | 9.0 |  | 6.7 |
|  | *H_e_* | **0.768*** | **0.596*** | 0.463 |  | **0.804*** | 0.840 |  | **0.769** |
|  | *F_IS_* | **0.236** | 0.422 | 0.237 |  | **0.235** | 0.087 |  | **0.289** |
| CxpGT20 | *A_R(26)_* | 15.3 | 13.8 | 8.3 |  | 14.4 | 12.5 |  | 14.4 |
|  | *H_e_* | 0.945 | 0.926 | 0.854 |  | 0.925* | 0.915 |  | **0.937** |
|  | *F_IS_* | 0.062 | 0.095 | 0.074 |  | **0.107** | 0.226 |  | **0.107** |
| CxpGT40 | *A_R(26)_* | 4.4 | 8.2 | 5.1 |  | 8.9 | 9.0 |  | 6.0 |
|  | *H_e_* | 0.425 | 0.718 | 0.514 |  | 0.513 | 0.712 |  | **0.619** |
|  | *F_IS_* | 0.176 | 0.111 | 0.110 |  | 0.070 | -0.004 |  | **0.262** |
| All loci | *A_R(26)_* | 8.6 | 8.2 | 5.1 |  | 8.9 | 9.0 |  | 9.0 |
|  | *H_e_* | **0.768** | **0.773** | 0.661 |  | **0.794** | 0.824 |  | **0.806** |
|  | *F_IS_* | **0.121** | **0.169** | 0.066 |  | **0.129** | 0.127 |  | **0.167** |

P: pipiens cluster; H: *admixed* individuals; M: molestus cluster; *A_R(26)_*: allelic richness for a minimum sample sizes of 26 genes (13 individuals); *H_e_*: expected heterozygosity; *F_IS_*: inbreeding coefficient. Values in bold indicate a significant *P*-value after correction for multiple tests (see Methods). Asterisks indicate presence of null alleles determined by Micro-Checker. Per locus and over sample Hardy-Weinberg tests were performed using ARLEQUIN. For over loci estimates the global test available in FSTAT was used.
